# Permeation dynamics of microemulsions according to the amount of deep eutectic solvent when applied to the stratum corneum†

**DOI:** 10.1039/d5ra00403a

**Published:** 2025-03-24

**Authors:** Shotaro Shinoda, Mina Tanigawa, Mina Sakuragi

**Affiliations:** a Faculty of Engineering, Department of Nanoscience, Sojo University 4-22-1 Ikeda, Nishi-ku Kumamoto City 860-0082 Japan d08b0101@nano.sojo-u.ac.jp

## Abstract

Deep eutectic solvents (DESs) can enhance the penetration of drug carriers in transdermal drug delivery systems. Previously, we showed that terpene-based DESs substantially enhance the penetration of drug carriers but cause skin damage. To retain the penetration-enhancing properties of DESs while mitigating their adverse effects on the skin, we incorporated small amounts of terpene-based DESs into the oil phase, formulating water-in-oil-type microemulsions (MEs). Stratum corneum (SC) lipid layers, which are sensitive to hydration levels, exhibit changes in spacing and regularity when interacting with DESs. Furthermore, DESs disrupt the lipid structure *via* unique mechanisms differing from those of traditional MEs. Herein, we investigated the effect of DES concentrations in the MEs on skin permeation under different hydration conditions. Utilizing synchrotron small-angle X-ray scattering and small-angle neutron scattering methods, we analyzed the molecular-scale interactions between the MEs and SC lipids to effectively understand their interaction behavior across hydration states. Overall, these findings highlight the importance of optimizing DES contents and SC hydration levels to achieve an efficient and safe transdermal drug delivery system.

## Abstract 2

The scattering techniques, including small-angle X-ray and neutron scattering, revealed how DES concentration and skin hydration affect stratum corneum lipid structure and drug carrier penetration.

## Graphical abstract

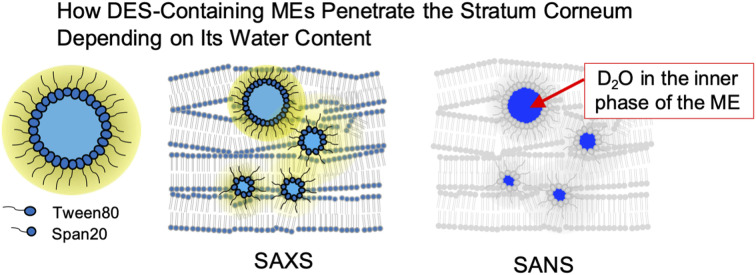

## Introduction

Transdermal drug delivery systems (TDDSs), which involve applying a patch to the skin and delivering a controlled dose of a drug or biomolecule, have gained attention because they are painless and can release a low drug dose over a long time. The primary rate limiter for TDDSs is the stratum corneum (SC), which is the outermost layer of the skin and comprises a lipid layer and corneocytes. It plays an important role in the barrier and water retention functions of the skin. Differential scanning calorimetry has shown that the water content of the SC is ∼33%.^1^ The SC water content affects the structural conditions of the lipid layer, such as its spacing and regularity,^2^ which in turn affect the penetration by TDDS carriers, such as water-in-oil-type microemulsions (W/O-type MEs).^3^ In our previous study, we assumed that the SC water content affects the skin penetration mechanism of a drug carrier because the carrier has to cross the intercellular lipid lamellae in the SC.^4^ We used synchrotron small-angle X-ray scattering (SAXS) and small-angle neutron scattering (SANS) to reveal the interaction mechanism between W/O-type MEs and the SC lipid layer and found that the MEs absorbed water from the SC when the SC was hydrated, which decreased the distance between repeating units (*i.e.*, repeat distance) of the lipid layer over time. The size and shape of MEs are closely related to their drug encapsulation capacity and skin permeation efficiency.^5,6^ In addition, as the skin layer targeted for drug or active ingredient delivery depends on the drug type and purpose, understanding the structural changes of MEs within the skin, such as loss of structural stability or disintegration in certain skin parts, is critical.^7,8^

In recent years, deep eutectic solvents (DESs) and ionic liquids (ILs) have gained attention for their ability to enhance the skin penetration of drug carriers by disrupting the structure of the SC lipid layer. ILs are organic salts comprising relatively large asymmetric organic cations and inorganic anions, and DESs are formed by the self-association of two or more components through hydrogen bonds.^9,10^ Although first-generation ILs based on imidazolium and phosphonium were too toxic for use in TDDSs, some biocompatible ILs have been developed in recent years.^11^ DESs can be applicable to TDDSs because of their advantages such as low costs, nontoxicity, easy preparation, and high biodegradability.^12–15^ ILs with higher hydrophobicity exhibit greater skin penetration-enhancing effects.^16^ Similarly, DESs with higher hydrophobicity enhance skin penetration more effectively.^17^ This is possibly because the SC, which serves as the rate limiter barrier for skin permeation, is inherently hydrophobic. In contrast, hydrophilic DESs exhibit skin penetration-enhancing effects depending on their composition. Geranic acid–choline DESs improve the penetration of protein and other drugs into the skin by partly extracting SC lipids.^18,19^ The composition of DESs influences the solubility of drugs and dispersion stability of drug carriers. Therefore, an appropriate DES composition must be selected based on the drugs and drug carriers. Unlike conventional skin penetration enhancers (*i.e.*, ethanol, l-menthol, fatty acids, DMSO, and so on), DESs offer flexibility in optimizing drug solubility and permeability by allowing control over physicochemical properties such as polarity and viscosity, through their composition.^20^

Terpenes such as menthol and thymol are good candidates for preparing sustainable and cheap hydrophobic DESs because of their low solubility in water and low prices.^21^ A DES comprising monocarboxylic acid and terpene dissolves risperidone 7300 times more effectively than water, resulting in enhanced skin permeability of risperidone.^22^ Additionally, thymol–decanoic acid is a hydrophobic DES that can enhance SC permeation of hydrophilic fluorescein sodium and hydrophobic meloxicam.^17^ X-ray scattering and IR studies have revealed that although thymol-based DESs extract some SC lipids and permeated corneocytes, they induce skin damage.^17^ Therefore, before utilizing thymol-based DESs as skin penetration enhancers, we must mitigate their irritant effect on the skin.

Herein, a small amount of a DES was dispersed in the oil phase of W/O-type MEs to reduce skin damage while maintaining the skin penetration effect. We investigated the structural changes that occur when an ME containing DES in the oil phase penetrates the SC depending on the SC water content. Because the DES alters the structure of SC lipid lamellae, we anticipated gaining novel insights distinct from the permeation mechanism of conventional W/O-type MEs. Clarifying the interaction between a drug carrier and SC lipids at the molecular level is essential for developing the TDDS field.

## Experimental

### Material

Tween-80 (oleic acid, ≥58.0% (with primarily linoleic, palmitic, and stearic acids)) and Span-20 (linoleic acid, ≥44.0% (with primarily myristic, palmitic, and linoleic acids)) were purchased from Sigma-Aldrich (Merck KGaA, Darmstadt, Germany) *via* Merck Ltd (Japan). Isopropyl myristate (IPM), rutin hydrate (>98.0%, determined *via* titration), and thymol (>99.0% *via* GC) were obtained from Tokyo Chemical Industry Co., Ltd (TCI, Tokyo, Japan). Decanoic acid (≥99.0% *via* GC), trypsin, and a trypsin inhibitor were purchased from FUJIFILM Wako Pure Chemicals (Osaka, Japan). Full-thickness skin of seven-week-old male hairless mice (Hos:HR-1, thickness: ∼0.7 mm) with the subcutaneous fat removed was purchased from Japan SLC, Inc.

### ME preparation

MEs were prepared by mixing water, two nonionic surfactants (Tween-80 and Span-20), and IPM or a mixture of IPM and a DES. Samples were sonicated using a probe sonicator (UH-50, SMT Co., Ltd). The weight ratio of Tween-80 and Span-20 was adjusted to 9 : 1, and the IPM–DES mixture was adjusted to 0 : 1, 1 : 9, 1 : 6, 1 : 4, and 1 : 0 ratios. The DES was prepared by mixing decanoic acid and thymol at a molar ratio of 2 : 3 and was heated in a water bath at 80 °C until a homogeneous liquid mixture was obtained. The weight ratio of the surfactants, water, and IPM was maintained at 20 : 4 : 76. Assuming that the DES acts as a surfactant, the hydrophilic–lipophilic balance (HLB) values of the combined surfactants and DES (HLB_mix_) were calculated using the Griffin–Davies equation, HLB_mix_ = *f*_A_HLB_A_ + *f*_B_HLB_B_ + *f*_C_HLB_C_,^23^ where HLB_A_, HLB_B_, and HLB_C_ represent the HLB values of Tween-80 (HLB = 15.0),^23^ Span-20 (HLB = 8.6),^23^ and DES, respectively, and *f*_A_, *f*_B_, and *f*_C_ denote their respective weight fractions. To calculate the HLB for the DES, those of decanoic acid and thymol were determined as 4.8 and 4.15, respectively, using Davies equations.^24^ These HLB values and mass fractions of decanoic acid and thymol in the DES were used to determine the HLB value of the DES as 4.4.

### Trans-epidermis water loss measurements

The full-thickness hairless mouse skin was incubated at room temperature and 50% RH for 30 min. The skin was then cut into small pieces of approximately 4 × 4 cm^2^. Each IPM–DES mixture was added to a skin sample in a glass Petri dish. After 3 h, the solution on the skin was removed by a laboratory wipe. The skin sample was then washed with 70% ethanol twice, with 20% ethanol seven times, and with distilled water twice. Excess water on the surface was gently wiped away after the skin samples were immersed in pH 7.4 phosphate buffer for 24 h, and the trans-epidermal water loss (TEWL) of each skin sample was measured using a Tewameter (TM Hex, Courage, and Khazaka Electronic GmbH, Cologne, Germany) at 50% RH.

### Dynamic light scattering measurements

DLS experiments were performed at The University of Kitakyushu using a DelsaMax Light Scattering Analyzer (Beckman Colter) at a wavelength of 532 nm and scattering angle of 90° with a 45 μL transparent cell having a length of 1 cm. All sample data were measured at a temperature of 25 °C and represented with a histogram. The average radius peaks for the formed dispersed system and aggregates were plotted.

### Skin penetration experiments

Skin samples were incubated at 10% RH for 1 h and 90% RH for 2 h. Skin penetration experiments were conducted using Franz diffusion cells (PermeGear, Inc., USA) with a diameter of 5.0 mm. A mixture solvent of pH 7.4 phosphate buffer, propylene glycol and ethanol in a 6 : 2 : 2 ratio was placed in the receptor chamber, which was continuously stirred at 600 rpm and maintained at 37 °C. Skin samples preincubated at each humidity level were placed onto the receptor chamber. Then, 50 μL of an ME containing 1.0 mg g^−1^ of rutin was applied to the SC surface. After 48 h, the concentration of rutin in the receptor chamber was analyzed *via* high-performance liquid chromatography using a Shimadzu CTO20A system equipped with an octadecyl-modified PVA gel column (Asahipak ODP504E; 4.6 mm inner diameter, 250 mm length, and 5 μm particle diameter) from Shodex (Japan) using a similar approach as described in a previous study.^7^ The detection wavelength for rutin was set at 360 nm. The mobile phase comprised 2.0% acetic acid, 27% acetonitrile, and 71% methanol with a flow rate of 0.50 mL min^−1^. The skin penetration was reported as mean ± standard deviation (SD) (*n* = 3). Significant differences among samples were determined based on a one-tailed unpaired Student's *t*-test.

### X-ray scattering

X-ray scattering measurements were conducted at beamline 40B2 of Spring-8, which is a synchrotron radiation facility in Japan. X-ray diffraction profiles were obtained using a Pilatus detector for SAXS and a flat panel for wide-angle X-ray scattering (WAXS). The X-ray wavelength was 0.1 nm, and the sample-to-detector distance (SDD) was set at approximately 2000 mm for SAXS and 80 mm for WAXS. SAXS was employed to examine the ME structure, with WAXS being employed to analyze the changes to the SC hydrocarbon packing structure when MEs were applied. Exposure times were 15 and 4 s for SAXS and WAXS, respectively. The skin samples were immersed overnight at 4 °C in a 0.1% trypsin solution prepared in a 10 mM phosphate buffer (pH 7.4). The SC was then separated from the skin by incubation at 37 °C for 4 h. Following separation, the SC was immersed in a 0.1% trypsin inhibitor solution and rinsed with water. To prepare the dry SCs, five layers of SC were stacked on a PEEK film and dried overnight in a vacuum pump. For hydrated SCs, the weight of the SC stack was recorded after drying, after which it was immersed in water for 4 h. The SC was then wiped with laboratory wipes to remove any excess water. The dry and hydrated SC samples were each placed on a PEEK film, which was then placed in a custom sample cell designed by the Special Research Laboratory at the University of Kitakyushu Faculty of Environmental Engineering (Machine Center). Initial measurements of the SCs were recorded, after which the MEs were applied to the SC. For WAXS, scattering patterns were captured initially at 1 min after ME application and subsequently at 5 min intervals for 180 min. For SAXS, the MEs were measured in a quartz cell capillary with a diameter of 2 mm.

### Small-angle neutron scattering

SANS measurements were performed to observe the structural changes in the internal phase of the MEs within the SC using the SANS-U spectrometer at the JRR-3 reactor of the Japan Atomic Energy Agency, Tokai, Japan. The SDD was set to 2 m, and the neutron wavelength was set to 0.7 nm with a wavelength distribution (Δ*λ*/*λ*) of 10% full width at half-maximum. The exposure time was 20 min, and all measurements were conducted at 25 °C. MEs for which H_2_O in the internal phase was replaced with D_2_O were denoted as d-MEs. For the hydrated SC, eight sheets of SCs on a PEEK film were immersed in H_2_O for 4 h. After any excess water was removed, d-ME penetration into the hydrated SC was measured. For the dry SC, eight sheets of SC were stacked and dried using a vacuum pump. Then, the SC sample was evaluated immediately after the d-MEs were applied.

### Fitting analysis of the SAXS and SANS profiles

The magnitude of the scattering vector is defined as *q* = 4π sin(*θ*/2)/*λ* (*θ* and *λ* represent the scattering angle and wavelength, respectively). To analyze the SAXS and SANS profiles, we fitted them using a scattering function of a sphere, as given by^25^
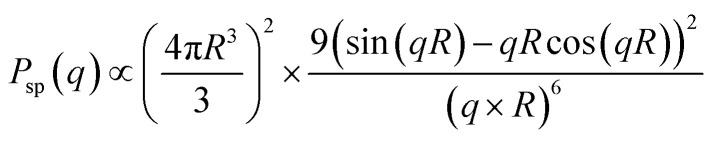
where *R* is the radius of a sphere, assumed to be exhibiting a Gaussian distribution.

## Results and discussion

**Fig. 1:**
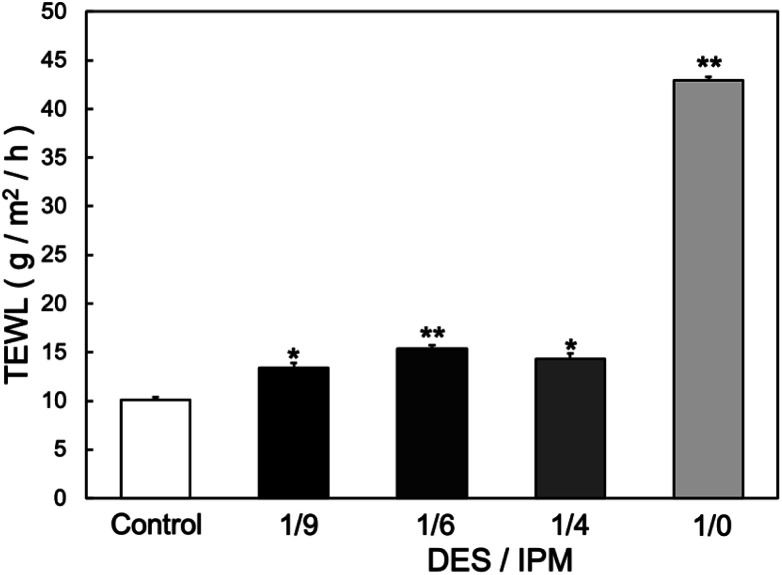
TEWL values at 24 h after applying each solvent to the skin (*n* = 3) *p < 0.05, **p < 0.01 *vs.* control.

W/O-type MEs were prepared by combining the water and oil phases of isopropyl myristate (IPM) with two nonionic surfactants: Tween-80 and Span-20. As the DES, thymol–decanoic acid was added to the oil phase. The weight ratio of the surfactants, water phase, and oil phase was fixed at 20 : 4 : 76. The weight ratio of Tween-80 and Span-20 was set to 9 : 1. Table 1 summarizes the code names, DES/IPM mixing ratios, HLB values, and structural properties (dispersion stability and sizes) of the prepared W/O-type MEs. The TEWL reflects the impairment of the skin barrier function, and adding a DES to the W/O-type MEs significantly decreased the TEWL from 44.1 ± 0.73 g m^−2^ h^−1^ for 1/0 ME to 13.4 ± 0.53 g m^−2^ h^−1^ for 1/9 ME (Fig. 1; *P* < 0.01), although 1/9 ME still showed a slight but statistically significant increase compared to the control (10.1 ± 0.26 g m^−2^ h^−1^; *p* < 0.05). Oudshoorn *et al.* reported that moderately and severely damaged skin exhibited TEWLs of 30 and ≥60 g m^−2^ h^−1^, respectively.^26^ Based on this criterion, MEs in our system exhibited mild skin irritation. However, further studies must be conducted to evaluate the long-term effects of repeated daily application of MEs containing DESs to ensure their safe prolonged use.

**Table 1:** Details of the prepared W/O-type MEs

Code name	DES/IPM (wt/wt)	HLB values	Stability^a^	Radius (nm)	
				SAXS^b^	SANS^c^
1/0 ME	1/0	6.5	×	—	—
1/4 ME	1/4	10.1	Δ	—	—
1/6 ME	1/6	10.9	○	5.1	4.3
1/9 ME	1/9	11.6	○	4.8	4.0
0/1 ME	0/1	14.4	Δ	—	—

a ○: stable for two weeks, Δ: separated within two days, and ×: separated within one day.

b Particle sizes were obtained *via* fitting analysis in the Guinier region.

c Sizes of the internal phase were obtained *via* fitting analysis.

In terms of stability, 1/6 ME and 1/9 ME remained dispersed for more than two weeks. MEs with a large amount of DESs (*e.g.*, 1/4 ME and 1/0 ME) were not stably dispersed. In addition, the ME with only IPM (*i.e.*, 0/1 ME) was not stably dispersed. In our previous study, MEs were stably dispersed in IPM when the weight ratio between Tween-80 and Span-20 was relatively balanced.^7,27^ The results of this research could be attributed to the assumption that the DES behaves as a surfactant, which resulted in 1/9 ME and 1/6 ME having HLB values close to that of IPM (11.1).^28^ In general, stable MEs are formed by ensuring that the surfactants and solvent have similar HLB values.^29^ These findings suggest that the DES acts as a surfactant within the MEs.

Synchrotron SAXS is an essential technique for investigating dynamic structural changes at the molecular level over a short time interval because it provides precise scattering profiles with minimal exposure time.^6,30^ Additionally, SANS can be utilized with H/D isotope substitution for the selective visualization of specific components. By integrating synchrotron SAXS with SANS, comprehensive and accurate structural insights into the intercellular lipids of the SC and drug carrier can be achieved. Herein, SAXS was employed to characterize the structures of 1/9 ME and 1/6 ME. The obtained profiles were fitted to the theoretical equation of a sphere with a Gaussian distribution (Fig. S1†). The particle radii of 1/6 ME and 1/9 ME were 5.1 and 4.8 nm, respectively. The larger particle size for 1/6 ME may be attributed to it containing a larger DES amount. The internal water phase of the MEs was replaced by deuterium to analyze the size of the internal phase, and the SANS profiles of these two MEs and their theoretical curves showed good agreement (Fig. S2†). The internal phases of 1/6 and 1/9 ME had radii of 4.3 and 4.0 nm, respectively. The particle size distributions of the MEs obtained *via* dynamic light scattering (Fig. S3†) and the percentages of aggregates present in the MEs (Table S1†) indicated that 1/6 ME and 1/9 ME had smaller amounts of aggregates compared to the other MEs.

**Fig. 2:**
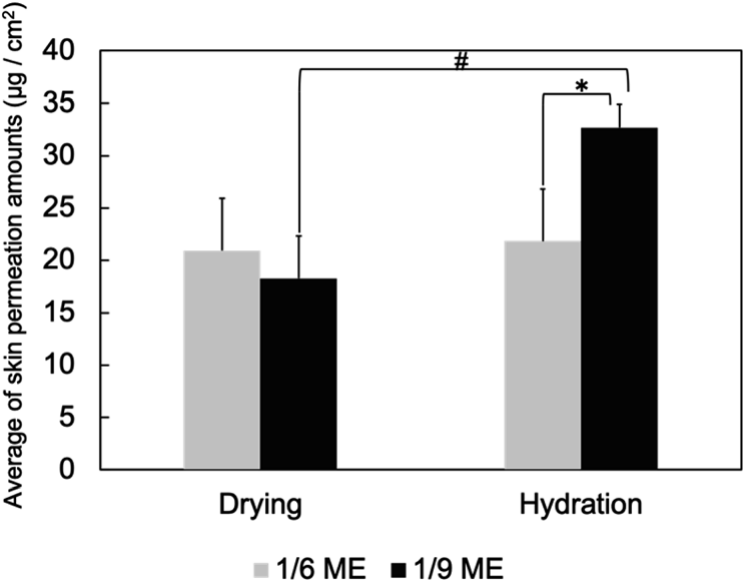
Skin penetration by MEs under dry and hydrated conditions. Data are presented as means ± SDs of three experiments. *p < 0.05 *vs.* 1/9 ME under dry conditions; #*p* < 0.05 *vs.* 1/6 ME under hydration conditions.

Subsequently, skin penetration tests were performed under different hydration conditions. Rutin, which is a flavonoid often used as an antioxidant in cosmetics^31^ and a therapeutic agent in medical applications, such as the prevention of cardiac remodeling,^32^ was used to label the MEs because it is insoluble in the internal water phase of the MEs and in IPM and DES but dissolves in the surfactants.^31,33^ Considering its log[octanol–water partition coefficient, *P*] (Log *P*) value (0.61)^34^ and its molecular weight (610), rutin cannot easily penetrate the skin without a drug carrier. This is because a Log *P* value between 1 and 3 (ref. 35) and molecular weight less than 500 (ref. 36) are associated with optimum skin permeation. Therefore, rutin is a suitable target for TDDS. With the rutin concentration used herein, no significant effects were observed on the structure or stability of the ME without rutin. Therefore, rutin was used as a label of ME herein. Fig. 2 presents the skin penetration results. Under the dry condition, 1/6 ME had slightly greater skin penetration than 1/9 ME, attributed to the former containing a larger DES amount that helped it disrupt more of the SC lipid layer. Under the hydrated condition, the skin penetration of 1/9 ME increased 1.8 times compared to under the dry, while the skin penetration of 1/6 ME showed almost no change. For comparison, the skin penetration amount of 0/1 ME (conventional W/O-type ME dispersed in IPM) was also evaluated under the dry condition, which yielded values of 2–14 μg cm^−2^ (data not shown). Although the permeation was lower than that of a DES-containing ME, the data exhibited significant variation and lacked reproducibility, probably due to its low dispersion stability. Control experiments in which rutin was directly dissolved in a solvent (IPM/DES) or water were not conducted because rutin did not dissolve in IPM, a DES, and water.

**Fig. 3:**
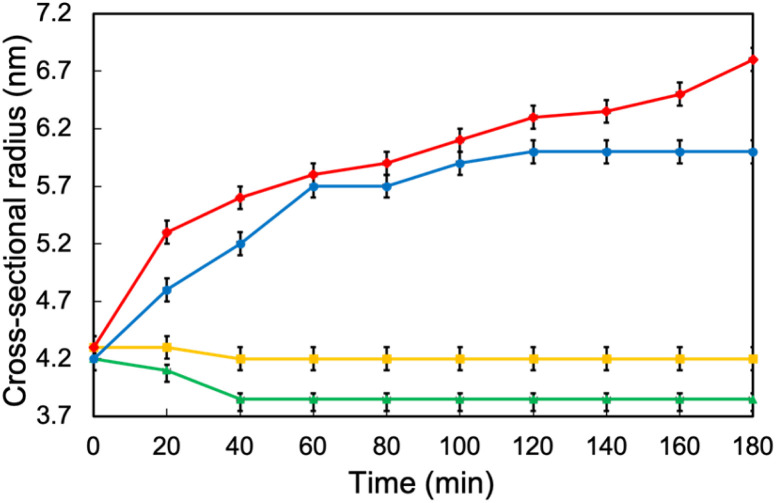
Size changes of the internal phases of d-MEs applied to dry and hydrated SCs over time (yellow: 1/6 ME applied to dry SC, green: 1/9 ME applied to dry SC, blue: 1/6 ME applied to hydrated SC, and red: 1/9 ME applied to hydrated SC).

**Fig. 4:**
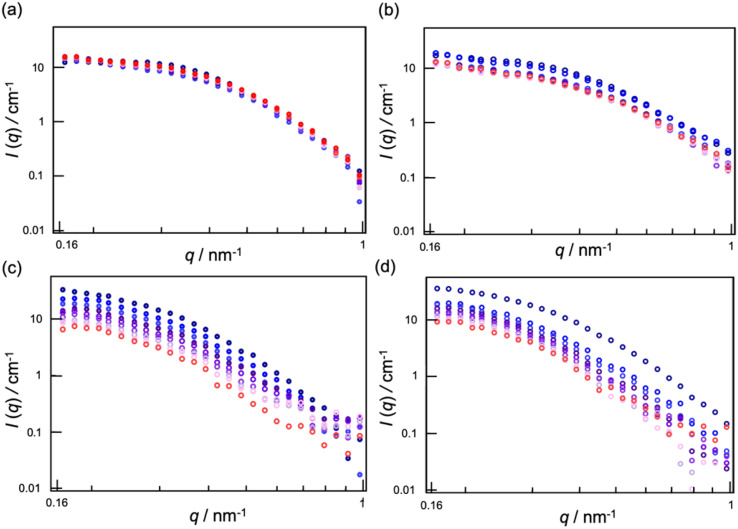
SANS profiles of (a) 1/6 d-ME applied to dry SC, (b) 1/9 d-ME applied to dry SC, (c) 1/6 d-ME applied to hydrated SC, and (d) 1/9 d-ME applied to hydrated SC. The color changed over time from blue to red.

Next, the structural changes to the MEs as they penetrated dry and hydrated SCs were evaluated using SANS. The internal water phase of the MEs was replaced with deuterated water (d-MEs). For the hydrated condition, d-MEs were evaluated after applying to the stacked SC that had been immersed in H_2_O for 4 h. For the drying condition, d-MEs were evaluated after applying to the SC that had been dried using a vacuum pump. Both 1/6 ME and 1/9 ME maintained spherical structures as they penetrated each SC. Fig. 3 shows the change in radii over time after the d-MEs were applied to each SC. On the dry SC, the radius of 1/6 ME only slightly decreased compared to its radius before SC application, whereas that of 1/9 ME decreased to a greater extent. The absolute-intensity SANS profiles of 1/6 ME remained almost constant over time (Fig. 4a), whereas the absolute intensity of 1/9 ME slightly decreased until 40 min (Fig. 4b), possibly because the size decreased as D_2_O was released from the inner phase of the MEs to the dry SC. On the hydrated SC, the absolute intensity of both MEs decreased (Fig. 4c and d), while the size of the internal phase increased over time. This is because the d-MEs absorbed H_2_O into their internal phases from the SC lipid lamellae, which led to incoherent scattering effects.^37^ The fluctuation at the high *q* region over time following d-ME application may also reflect the influence of incoherent scattering effects from H_2_O. The increase in particle size of 1/9 ME reached saturation after 2 h, whereas the particle size of 1/6 ME continued to increase even after 3 h. This may be because 1/6 ME contained a larger DES amount, and the DES and SC lipids that it extracts increase the ME particle size by acting as surfactants. The particle size of 1/6 ME increased within SC with a high-water content, which may have hindered its permeation through the SC and supports the results regarding the skin penetration amounts (Fig. 2).

The SC lipid layer has two types of repeating units: short lamellae (S-La) with a repeat distance of ∼6 nm and long lamellae (L-La) with a repeat distance of 13.6 nm. S-La has a water phase, while L-La does not. The hydrocarbon chains in L-La are packed in a hexagonal phase (Hex) with a lattice spacing of 0.41 nm. The hydrocarbon chains in S-La are packed in an orthorhombic phase (OR) with lattice spacings of 0.37 and 0.41 nm.^38^ For the SAXS measurements, the peaks of the SC lamellae overlapped with the form factor of the MEs, which made it difficult to analyze their structures. However, the peaks of the hydrocarbon chains could be observed in the high *q* region and did not overlap with the form factor of the MEs. Two peaks at *d* = 0.37 and 0.41 nm were observed after ME application (Fig. S4†).

**Fig. 5:**
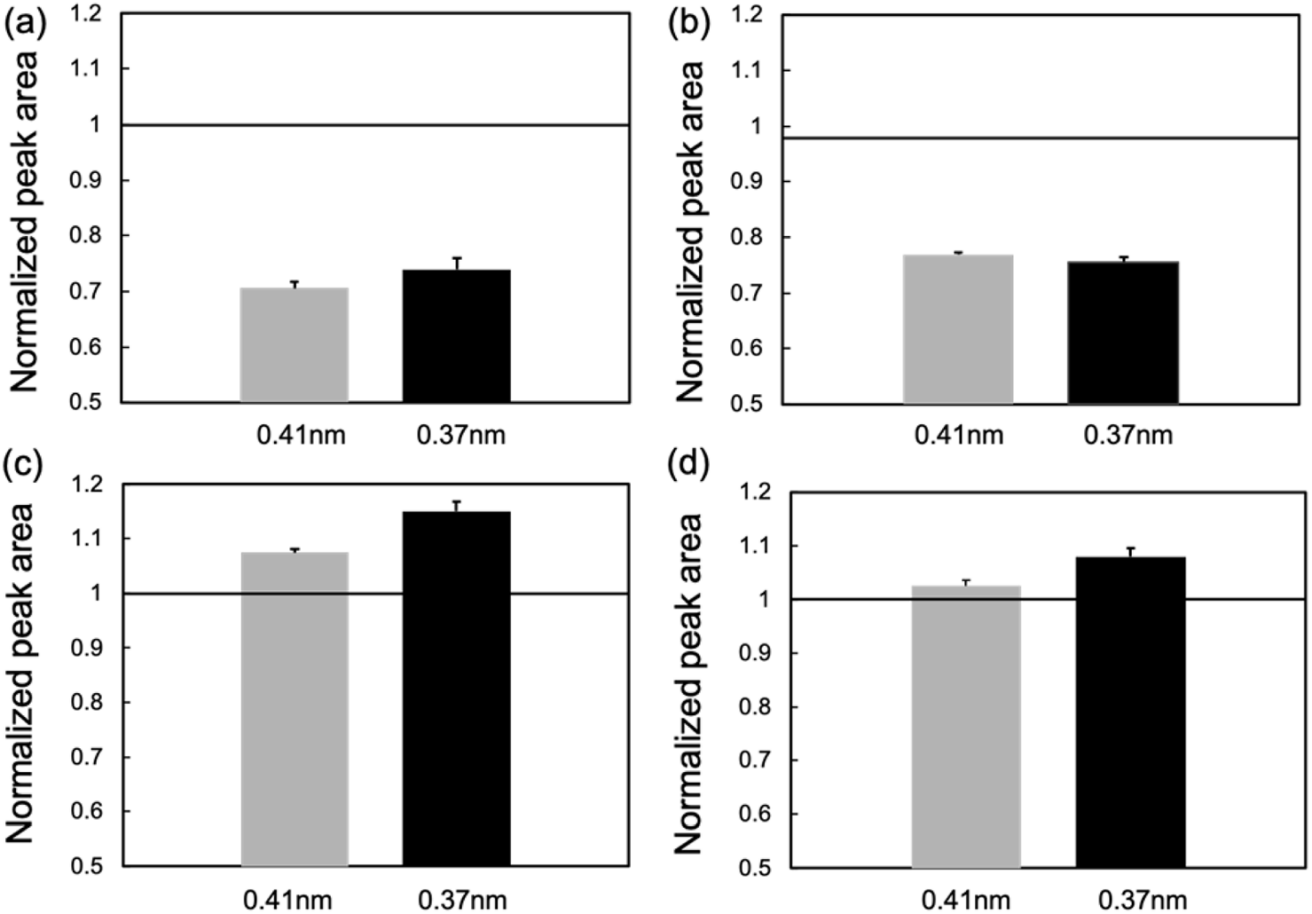
Normalized peak-area ratios of *d* = 0.41 and 0.37 nm at 180 min to 1 min after applying (a) 1/6 ME to dry SC, (b) 1/9 ME to dry SC, (c) 1/6 ME to hydrated SC, and (d) 1/9 ME to hydrated SC (*n* = 6).

Fig. 5 shows the normalized peak areas of Hex and OR at 180 min to 1 min after the MEs were applied to dry and hydrated SCs. When the MEs were applied to the dry SC, the peak area tended to decrease because the packing structure of the lipid layer was disrupted by ME penetration. The peak areas were smaller for 1/6 ME than for 1/9 ME.

Fig. S4† shows that the peak positions for both MEs did not change after 3 h of application to the dry SC. This indicated that the repeat distances of Hex and OR remained intact even if MEs penetrated the SC. A previous study wherein IR and SAXS were used revealed that DESs containing thymol–decanoic acid extracted SC lipids due to strong interactions between the hydrocarbon chains of DESs and SC lipids.^17^ These findings were consistent with the study wherein a hydrophobic DES was used as a skin penetration enhancer.^39^ These results demonstrated that lipids in the regions where the ME permeated were partly extracted, causing structural defects in the ordered arrangement of SC lipids, while the fluidity of SC lipids that were not extracted remained unchanged.

When the MEs were applied to the hydrated SC, the peak areas increased. The regularity of the hydrocarbon chain packing structure is known to be lower under dry (low RH) and hydrated (100% RH) conditions than at 50–95% RH.^40^ Herein, similar to the previous paper, we observed that the hydrocarbon chain packing structure exhibited lower regularity under high hydration or dry conditions, while the packing became more ordered when the SC contained a moderate amount of water (Fig. S5a and b†). Accordingly, the increase in peak areas with hydrated SCs means that the internal phase of the MEs absorbed water, which decreased the water content of the SCs. The increase in peak intensity was more pronounced for 1/6 ME than for 1/9 ME, indicating that 1/6 ME absorbed more water. By comparing the peak area in Fig. S5† with that in Fig. 5, the SC water content when applying 1/6 ME and 1/9 ME can be estimated as approximately 40% and 70%, respectively. The ME did not possibly affect the fluidity of the hydrated SC lipids, as well as dry SC, because the peak positions did not change between 1 min and 3 h after applying MEs.

**Fig. 6:**
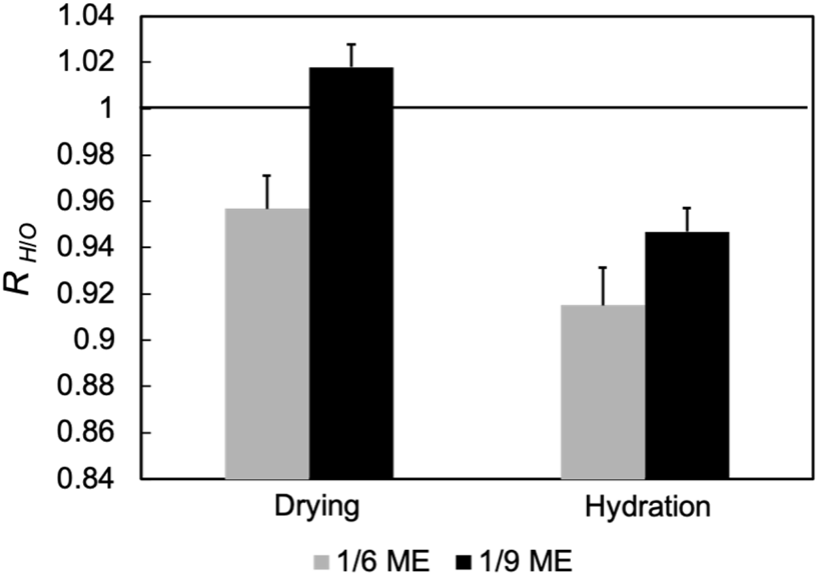
Normalized *R*_H/O_ at 180 min to 1 min after applying MEs.

To determine whether Hex or OR was affected, the apparent abundance ratio *R*_H/O_ was defined as follows:^13,41,42^
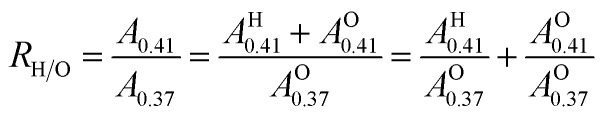
where *A* indicates the peak area, the subscripts 0.41 and 0.37 indicate the lattice spacing (*d*), and the superscripts O and H indicate OR and Hex, respectively. The first term indicates the Hex–OR ratio, whereas the second term is a constant value. Fig. 6 shows the *R*_H/O_ ratios at 180 min to 1 min after the MEs were applied. For the dry SC, *R*_H/O_ increased for 1/9 ME, while it decreased for 1/6 ME, which means that 1/6 ME primarily disrupted Hex over OR while 1/9 ME mainly disrupted OR. Because the hydrocarbon chains were packed in Hex for L-La and in OR for S-La, 1/6 ME mainly penetrated L-La while 1/9 ME mainly penetrated S-La. As shown in Table 1, 1/6 ME has a lower HLB value and is more hydrophobic than 1/9 ME, facilitating its penetration into more hydrophobic L-La. As L-La has no water phases, 1/6 ME likely retained its water and thus exhibited a smaller particle size reduction rate than 1/9 ME.

For the hydrated SC, *R*_H/O_ decreased for both MEs. Considering that the peak areas increased over time after ME application (Fig. 6), the decrease in *R*_H/O_ indicates that the MEs preferred the ordering of OR. Applying 1/6 ME resulted in a more ordered OR structure than applying 1/9 ME. As shown in Fig. S5c,† the OR structure became preferentially more organized with decreasing water content in the SC, reaching its most organized state at a water content of ∼20%. In hydrated SC, 1/6 ME, which absorbs more water than 1/9 ME, more effectively enhances OR organization. Accordingly, the MEs disrupt the hydrocarbon packing structures of the dry SCs and absorb water from the hydrated SC, thus altering the SC water content.

In our previous study,^4^ we observed that conventional W/O-type MEs without a DES decrease the regularity of the SC lipids in hydrated SCs. Moreover, SANS analysis indicated that the particle size increases over time as water in the SC is absorbed in the inner phase of the MEs. After >2 h of application, the intensity of the scattering profiles decreased and the experimental curves deviated from the theoretical curves of a sphere. In the previous paper, we ascribed this phenomenon to incoherent scattering caused by H_2_O incorporation into the inner phase of the MEs. However, it might also be explained by the partial collapse of the ME structures, specifically involving the disruption of the surfactant-stabilized interface, and the subsequent release of water to the SC. Therefore, in our previous paper, the application of W/O-type MEs to the hydrated SC probably disrupted the packing structure of the SC lipids. In contrast, herein, the DES-containing MEs maintained their structure even after 3 h of application, increasing their sizes by absorbing water from the SC. The surrounding DES structures stabilized the ME structures during SC penetration. The corresponding results are summarized in Table 2. These findings are a major contribution of our current research.

**Table 2:** Summary of the obtained results

SC condition	Dry SC		Hydrated SC	
Code name	1/6 ME	1/9 ME	1/6 ME	1/9 ME
Skin permeation amounts (μg cm^−2^)	20.90 ± 3.16	18.24 ± 4.08	21.82 ± 2.95	32.69 ± 2.24
Changes in the inner radius of ME in the SC	4.3 nm → 4.2 nm (3 h)	4.0 nm → 3.85 nm (3 h)	4.3 nm → 6.8 nm (3 h)	4.0 nm → 6.0 nm (3 h)
	Slightly decreased	Decreased	Constantly increase even after 3 h	Constantly increased until 2 h
Changes in normalized peak-area ratios of SC lipid packing structures	0.71 ± 0.012 (*d*_0.41_)	0.77 ± 0.0043 (*d*_0.41_)	1.07 ± 0.0067 (*d*_0.41_)	1.02 ± 0.012 (*d*_0.41_)
	0.74 ± 0.020 (*d*_0.37_)	0.76 ± 0.0095 (*d*_0.37_)	1.15 ± 0.021 (*d*_0.37_)	1.08 ± 0.012 (*d*_0.37_)
	Regularity decreased	Regularity decreased	Regularity increased	Regularity increased
*R*_H/O_	0.96 ± 0.014	1.02 ± 0.012	0.92 ± 0.019	0.95 ± 0.012
	Hex is more disrupted than OR	OR is more disrupted than Hex	OR is more ordered than Hex	OR is more ordered than Hex

## Conclusions

Herein, W/O-type MEs were prepared by dispersing varying amounts of a DES in the oil phase, aiming to enhance skin penetration while minimizing potential damage. Among the prepared MEs, 1/6 ME and 1/9 ME demonstrated stable dispersion for at least 2 weeks and were subsequently employed to evaluate their skin penetration performance and structural interactions with the SC under dry and hydrated conditions.

When applied to dry SCs, 1/6 ME exhibited a greater skin penetration than 1/9 ME, although the difference was not statistically significant. In addition, 1/6 ME tended to disrupt the packing structure of the SC hydrocarbon chains more effectively than 1/9 ME with a particular tendency to preferentially disrupt L-La, possibly owing to a lower HLB value of 1/6 ME. When applied to hydrated SCs, 1/9 ME demonstrated a significant increase in skin penetration compared to 1/6 ME despite the lower DES content. SANS results showed that 1/6 ME increased in size more than 1/9 ME over time because DES and the SC lipids it extracted acted as a surfactant. The large size of 1/6 ME may reduce the skin penetration amount. In addition, under the hydrated condition, 1/6 ME organized the packing structure of the SC hydrocarbon chains more regularly than 1/9 ME, which was attributed to the former absorbing water from the SC in its internal phase. Thus, the lack of change in skin penetration by 1/6 ME when applied to dry and hydrated SCs could be attributed to its size increase and the enhanced regularity of the SC packing structure.

Overall, 1/9 ME demonstrated superior characteristics (dispersion stability, low skin irritation, and permeability) when applied to the skin under hydrated conditions. In contrast, 1/6 ME might be a more suitable formulation for individuals with dry skin. Overall, the insights gained from this research provide a foundational framework for optimizing the amount of DES and SC water contents to improve the skin penetration ability of MEs, realizing MEs with high skin-penetration efficiency, a stable formulation, and skin safety. These findings enhance our understanding of the interactions between DES-containing MEs and SC lipids, which are influenced by the skin hydration levels and condition of an individual, and can promote the advanced design and development of effective TDDSs for clinical application. Future research should focus on optimizing the administration method of MEs to the skin *in vivo*, such as prehydration of the skin or application to the skin with different barrier conditions, which are essential for developing clinically relevant formulations. Additionally, a potential challenge that needs to be addressed is the evaluation of possible skin damage and its extent upon daily application of MEs, although MEs in our system did not exhibit severe skin damage in TEWL measurements *in vitro*. As rutin was used as the model drug herein, further investigation is required to generalize these findings to other drugs.

## Data availability

All data generated or analyzed during this study are included in this published article and its ESI files.†

## Author contributions

SS: data curation, experiments, structural analysis, investigation, methodology, writing – original draft. MT: experiments, writing – review and editing. MS: investigation, methodology, funding acquisition, project administration, resources, supervision, writing – original draft, review, and editing.

## Conflicts of interest

There are no conflicts to declare.

## Supplementary Material

RA-015-D5RA00403A-s001
