# Supplementary material for: Permeation dynamics of microemulsions according to the amount of deep eutectic solvent when applied to the stratum corneum
Source: RSC Adv. 2025 Mar 24;15(12):8977–85. doi: 10.1039/d5ra00403a (PMC11931507; doi:10.1039/d5ra00403a)
Supplement: RA-015-D5RA00403A-s001 [file RA-015-D5RA00403A-s001.pdf]

Supplementary Information (SI) for RSC Advances.
This journal is © The Royal Society of Chemistry 2025

Supporting Information

Permeation dynamics of microemulsions according to the amount of deep eutectic solvent when applied to the stratum corneum

Shotaro Shinoda,[a] Mina Tanigawa[a] and Mina Sakuragi *[a]

[a]Faculty of Engineering, Department of Nanoscience, Sojo University, 4-22-1 Ikeda, Nishi-ku, Kumamoto City 860-0082, Japan

*Corresponding author
Mina Sakuragi, Associate professor
E-mail: d08b0101@nano.sojo-u.ac.jp
Tel: +8196-326-3593

<u>Table of Contents</u>

1. Results and Discussion

1-1. SAXS profiles of the MEs

1-2. SANS profiles of the d-MEs

1-3. DLS measurements of MEs

1-4. WAXS peaks of hydrocarbon chain packing in SCs when MEs were applied

1-5. WAXS peaks area depending on the SC water contents

2. Reference

1. Results and Discussion

1-1. SAXS profiles of the MEs

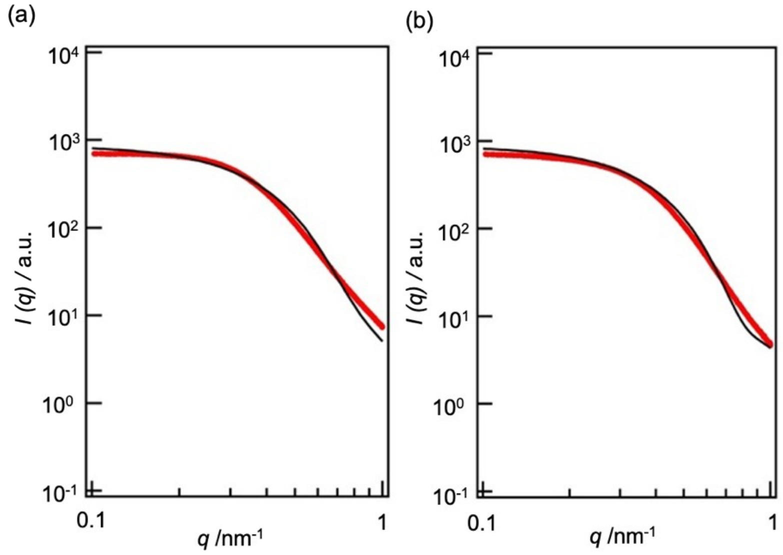

Fig.S1 SAXS profiles of a) 1/6 ME and b) 1/9 ME. Red: experimental data. Black: theoretical curve.

The experimental and theoretical curves exhibited good agreement in the low q region; however, discrepancies were observed in the high q region. The increased slope was attributed to the surfactant hydrocarbon chains behaving as Gaussian chains[1,2]. In this study, only the size and shape of the MEs were evaluated.

1-2. SANS profiles of the d-MEs

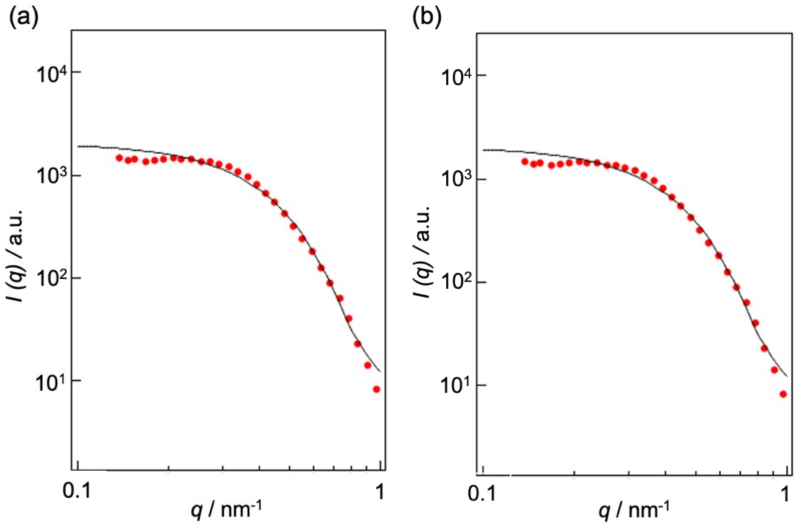

Fig. S2 SANS profiles of (a) 1/6 d-ME and (b) 1/9 d-ME (red: experimental data, black: theoretical curve).

The internal water phase of the MEs was replaced with D_2O to obtain d-MEs. The internal phases of 1/6 d-ME and 1/9 d-ME had radii of 4.3 and 4.0 nm, respectively.

1-3. Dynamic light scattering measurements of MEs

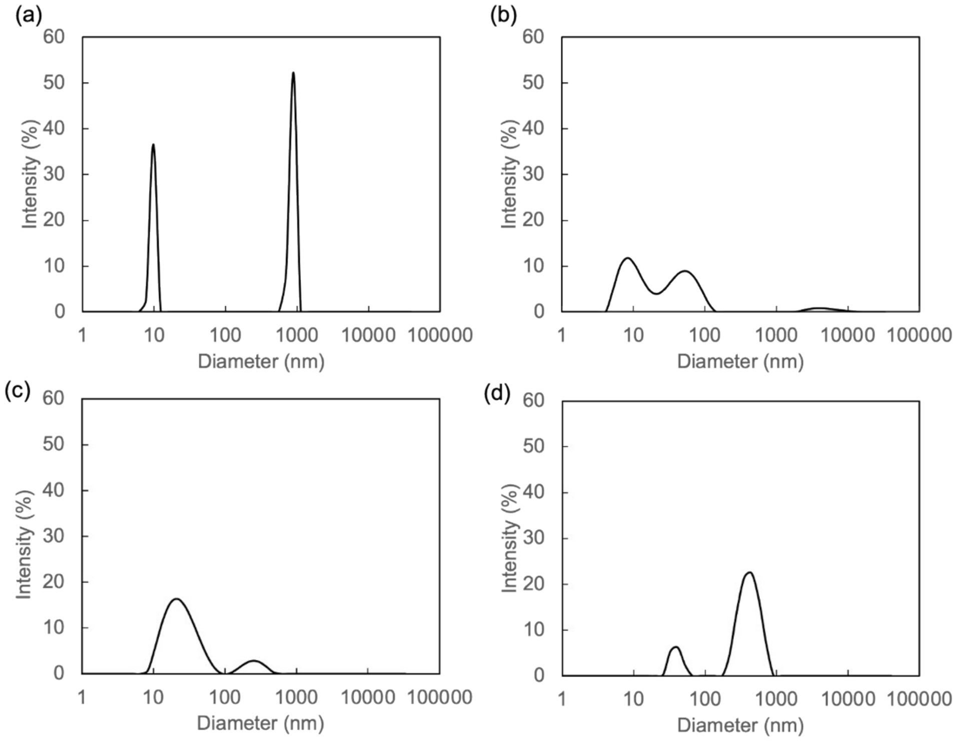

Fig. S3 Size distribution results for (a) 1/4 ME, (b) 1/6 ME, (c) 1/9 ME, and (d) 0/1 MEs obtained by DLS measurements at room temperature.

Table S1 Particle size distributions of MEs.

Sample name	Less than 100 nm (%)	More than 100 nm (%)
1/4 ME	39.2	60.8
1/6 ME	94.4	5.6
1/9 ME	89.5	10.5
0/1 ME	14.1	85.9

1/6 ME and 1/9 ME had fewer aggregates whereas 1/4 ME and 0/1ME exhibited some aggregation.

1.4. WAXS peaks of hydrocarbon chain packing in SCs when MEs were applied

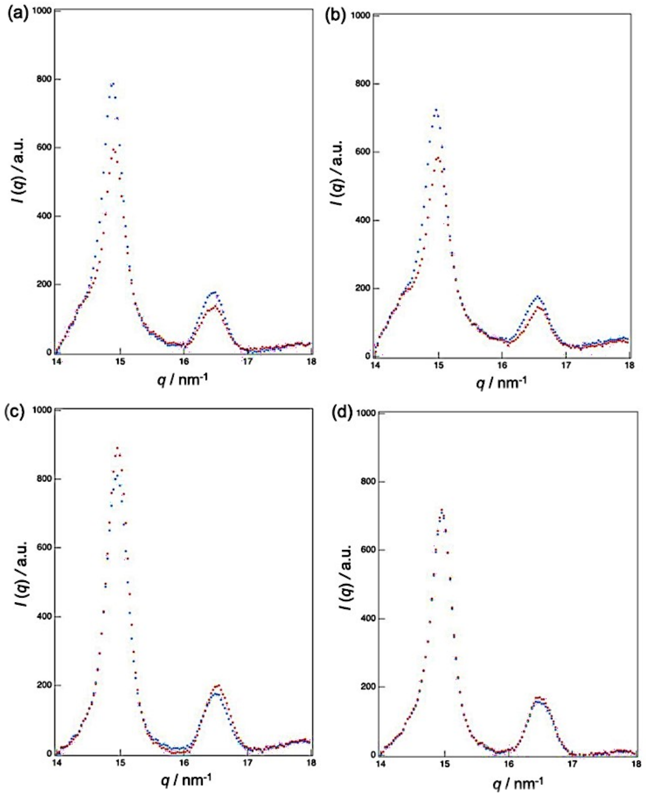

Fig.S4 WAXS profiles of SCs at 1 min (blue) and 180 min (red) after (a) 1/6 ME was applied to dry SC, (b) 1/9 ME was applied to dry SC, (c) 1/6 ME was applied to hydrated SC, and (d) 1/9 ME was applied to hydrated SC.

1-5. WAXS peaks area depending on the SC water contents

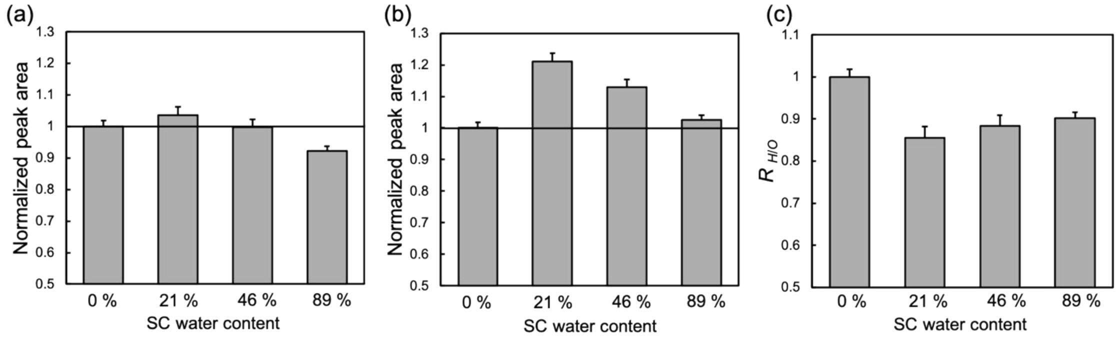

Fig. S5 Peak area ratios of (a) the 0.41 nm and (b) 0.37 nm peaks and (C) normalized $R_{H/O}$ values at each water content of the SC relative to those at 0% water content.

2. References

1 M. Sakuragi, S. Tsutsumi and K. Kusakabe, Langmuir, 2018, 34, 12635–12641.

2 J. S. Pedersen and M. C. Gerstenberg, Macromolecules, 1996, 29, 1363–1365.
